# Micronuclei Assessment of The Radioprotective Effects of Melatonin and Vitamin C in Human Lymphocytes

**DOI:** 10.22074/cellj.2016.3986

**Published:** 2016-04-04

**Authors:** Aram Rostami, Seyed Akbar Moosavi, Hassan Dianat Moghadam, Eftekhar Rajab Bolookat

**Affiliations:** 1 Department of Medical Physics, School of Medicine-International Campus, Iran University of Medical Sciences, Tehran, Iran; 2 Department of Lab Sciences, School of Allied Medicine, Iran University of Medical Sciences, Tehran, Iran; 3 Department of Medical Biotechnology, School of Advanced Technology, Tehran University of Medical Sciences, Tehran, Iran; 4 Department of Radiotherapy, Hospital of Shohada Tajrish, Shahid Beheshti University of Medical Sciences, Tehran, Iran

**Keywords:** Radioprotective, Melatonin, Vitamin C, Lymphocyte, Micronucleus

## Abstract

**Objective:**

Critical macromolecules such as DNA maybe damaged by free radicals that are generated from the interaction of ionizing radiation with biological systems. Melatonin and vitamin C have been shown to be direct free radical scavengers. The aim of this study was to investigate the *in vivo*/*in vitro* radioprotective effects of melatonin and vitamin C separately and combined against genotoxicity induced by 6 MV x-ray irradiation in human cultured blood lymphocytes.

**Materials and Methods:**

In this experimental study, fifteen volunteers were divided into three groups of melatonin, vitamin C and melatonin plus vitamin C treatment. Peripheral blood samples were collected from each group before, and 1, 2 and 3 hours after melatonin and vitamin C administration (separately and combined). The blood samples were then irradiated with 200 cGy of 6 MV x-ray. In order to characterize chromosomal aberrations, the lymphocyte samples were cultured with mitogenic stimulus on cytokinesisblocked binucleated cells.

**Results:**

The samples collected 1hour after melatonin and vitamin C (separately and combined) ingestion exhibited a significant decrease in the incidence of micronuclei compared with their control group (P<0.05). The maximum synergic protection and reduction in frequency of micronuclei (57%) was observed 1 hour after vitamin C and melatonin administration combined.

**Conclusion:**

We conclude that simultaneous administration of melatonin and vitamin C as radioprotector substances before irradiation may reduce genotoxicity caused by x-ray irradiation.

## Introduction

Irradiation due to radiotherapy or natural and industrial sources can damage the human tissues considerably (1). It has been recognized that the most critical target for transferring ionizing radiation in living tissue is the DNA (genetic material) that exists in the nucleus and mitochondria of most cells (2). Ionizing radiation generates reactive oxygen species (ROS) in irradiated cells. The free radicals such as hydroxyl (˚OH) and active hydror gen (˚H) can induce damage to the DNA in cells. Mutations and malignant lesions may be caused by these cellular DNA damages (3). Radiationinduced cellular damages are attributed mainly to the harmful effects of the free radicals. Molecules with the ability to sweep free radicals are thus useful, particularly as radioprotectors (4). Melatonin (N-acetyl-5methoxytryptamine) and Vitamin C are two well-known and established radioprotectors (5). Melatonin is an endogenous compound that is synthesized by the pineal gland in the human brain (6,8). It was shown that melatonin is a direct free radical scavenger (9). Melatonin is also an indirect antioxidant agent by stimulating antioxidant enzyme activity and inhibiting prooxidative enzyme activity (10,12). Many studies have indicated that both acute and chronic toxicity of melatonin is negligible. For instance, no negative side effects were observed in human volunteers orally ingesting melatonin at a dose of 1-300 mg or 1g for 30 days (13). Antioxidant vitamins have been also studied extensively for their capability of protecting human cells against induced damages by irradiation (14). It is well known that antioxidant vitamins such as vitamin C can protect cellular DNA and membranes from radiationinduced damages. The radioprotective effect of vitamin C is through scavenging molecular oxygen, hydroxyl radicals and atomic oxygen radicals (5). Chromosomal damages were assessed in this study by micronuclei analysis that is a powerful method in human biomonitoring of *in vivo* genotoxin exposure. Micronuclei may originate from either acentric chromosomal fragments or whole chromosome delay in the anaphase stage. This assay has been used widely to investigate the effects of potential radioprotector agents (15). 

Although many *in vitro* studies have shown that treatment of human peripheral blood lymphocytes with melatonin and vitamin C reduces the micronuclei induced by gamma irradiation (4,5), the efficacy of this treatment is yet to be evaluated *in vivo* by oral ingestion. In this study, we therefore evaluated the efficacy of the radioprotective effects of melatonin and vitamin C separately and combined against 6 MV x-ray radiation on human blood lymphocytes. 

## Materials and Methods

This experimental study was approved by the Ethics Committee of Iran University of Medical Sciences. Informed consent was obtained from fifteen healthy and non-smoking male volunteers with an average weight of 65 kg and mean age of 26 ± 3 years. The selected individuals had no history of pharmacotherapy for at least two months before sampling and did not suffer from any serious acute or chronic illness. The volunteers were divided into three groups, namely A, B and C. After overnight fasting, five volunteers (group A) were given a single oral dose of two gelatin capsules each containing 300 mg melatonin (Natrol® company), five volunteers (group B) were given two capsules each containing 300 mg of vitamin C (Baker England) and five volunteers (group C) were given a combination of both melatonin (one capsule) and vitamin C (one capsule). Blood samples were collected in heparinized tubes before (0 hour), and 1, 2, and 3 hours after melatonin (group A), vitamin C (group B) and melatonin plus vitamin C (group C) ingestion. 

For each volunteer at each sampling time, 1 ml of heparinized blood was divided into two halves and poured into two 25 ml culture flasks. One culture flask was kept as a non-irradiated control sample and another one was irradiated with a 6 MV x-ray accelerator unit (Oncor, Siemens) at 37˚C. The accelerator was calibrated for irradir ating human whole blood samples. To assess the calibration of the accelerator, a 200 cGy test run was performed on a tube filled with water and accuracy of dose distribution was estimated at about 4%. To measure radiation dose, thermoluminescent dosimeter (TLD) was used and after exposure, the TLDs were read by HARSHAW TLD 3500 Manual Reader. Obtained results of TLDs indicated that radiation dose has an accuracy of 4% were 200.04, 196 and 200.02 cGy for the top, bottom and the middle of the test tube respectively, for 200 cGy irradiation (16). After, irradiation was performed with a dose of 200 cGy at 100 cm SSD, 10 cm^2^field size and 0.731 cGy per monitor unit (MU) output factor. 

Subsequently, each sample (non-irradiated and irradiated) was added to 4.5 ml of RPMI 1640 culture medium (Sigma, USA) containing 20% fetal calf serum, 100 µl/ml phytohaemagglutinin (Sigma, USA), 100 μl/ml penicillin, 250 µg/ml streptomycin and 2 mM glutamine (Sigma, USA). All cultures were incubated at 37 ± 1˚C in a humidie fied atmosphere of 5% CO_2_and 95% O_2_. After 48 hours of culture, Cytochalasin B (Sigma Aldrich, final concentration: 30 µl/ml) was added to the samples. After 72 hours of incubation, cells were collected by centrifuging at 800 rpm for 5 minutes. 

The collected cells were suspended again in 0.075 M cold potassium chloride (KCl) and centrifuged at 800 rpm for 6 minutes and instantly fixed in a fixative solution (methanol: acetic acid, 6:1) for 3 times. To prepare the microscopic slides, the fixed cells were dropped onto clean microscopic slides, air-dried and stained with Giemsa solution. Assessment of slides was carried out at ×1000 magnification to determine the numbers of micronuclei in cytokinesis-blocked binucleate cells with the cytoplasm remaining intact. To score small nuclei as such, the following criteria were considered: diameter ranging from 1/16 to 1/3 of the diameter of the main nuclei, being non-refractive, no link or overlap with the main nuclei. For every volunteer and at each blood sampling time, for irradiated and control samples, 1000 cells were evaluated to score the frequency of micronuclei. 

### Statistical analysis

The student t test was used to compare incidence of micronuclei in each time point (1, 2, 3 hours) in three groups (A, B and C) with the irradiated control point (at 0 hour). The level of statistical significance was set to P<0.05. 

## Results

**Fig.1:**
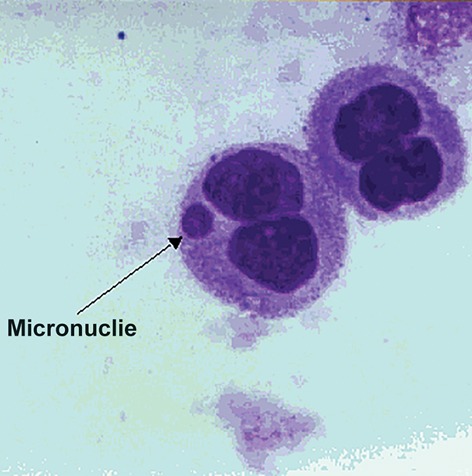
A typical binucleated lymphocyte with micronuclei.

We observed no side effects after the single oral ingestion of melatonin and vitamin C (separately and combined). Tables 1, 2 and 3 show a significant increase for the incidence of micronuclei in irradiated lymphocytes compared with the control cells without irradiation. A typical micronucleated binucleate cells shown in Figure 1. 

The percentage of micronuclei induced by irradiation in group A lymphocytes after 1, 2 and 3 hours of melatonin ingestion was 5.86 ± 2.32, 8.67 ± 1.24 and 10.28 ± 0.9, respectively. The frequency of micronuclei found in melatonintreated cell groups was significantly lower than the control group. For the blood samples collected 1 hour after the oral melatonin ingestion and exposed *in vitro* to 200 cGy of 6 MV radiation, a significant decrease in the incidence of micronuclei was observed when compared with corresponding samples collected at 0 hour (P<0.001). The incidence of total micronuclei was reduced to 55% at 1 hour after oral melatonin ingestion. Total micronuclei were typically lower at 1 hour compared with those at 2 and 3 hours after oral melatonin ingestion (Table 1). 

The percentage of the micronuclei induced by irradiation in group B human lymphocytes after 1, 2 and 3 hours of vitamin C administration was 7.13 ± 1.9, 9.86 ± 1.11 and 10.80 ± 0.6 respectively. The frequency of micronuclei found in vitamin Ctreated cell groups was significantly lower than the control group. For the blood samples collected 1 hour after oral vitamin C ingestion and exposed *in vitro* to 200 cGy of 6 MV x-ray radiation, a significant decrease in the incidence of micronuclei was observed when compared with corresponding samples collected at 0 hour (P<0.001). Total micronuclei were reduced to 65% at 1 hour after oral vitamin C administration. The incidence of total micronuclei was typically lower at 1 hour compared with those at 2 and 3 hours after oral vitamin C ingestion (Table 2). The percentage of the micronuclei induced by irradiation in group C human lymphocytes after 1, 2 and 3 hours of simultaneous melatonin and vitamin C administration was 4.66 ± 2.7, 7.90 ± 1.9 and 9.10 ± 0.9 respectively. The frequency of micronuclei found in melatonin and vitamin Ctreated cell groups was significantly lower than the control group. For the blood samples collected 1hour after oral melatonin plus vitamin C ingestion (combined) and exposed *in vitro* to 200 cGy of 6 MV x-ray radiation, a significant decrease in the incidence of micronuclei was observed when compared with corresponding samples collected at 0 hour (P<0.001). Total micronuclei were decreased to 43% at 1 hour after melatonin+vitamin C ingestion (combined). The incidence of total micronuclei was typically lower at 1 hour when compared with those at 2 and 3 hours after oral melatonin plus vitamin C ingestion (combined) as shown in Table 3. 

**Fig.2:**
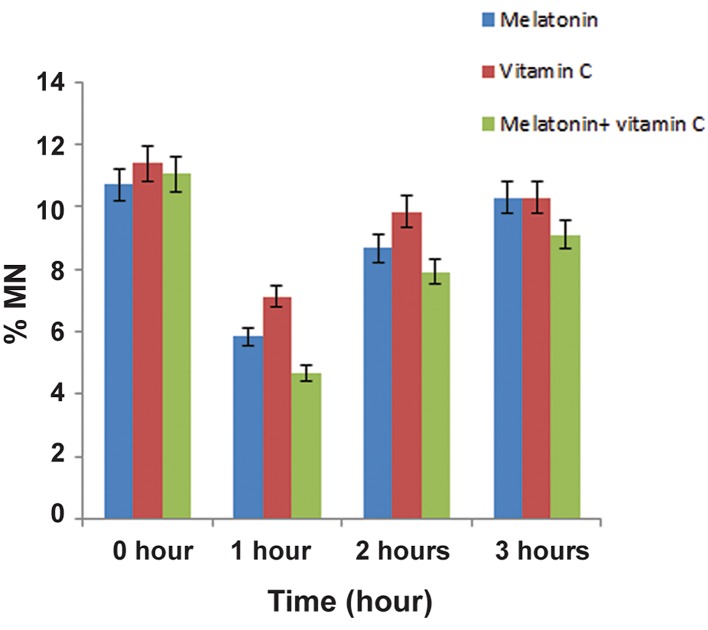
The average percentages ± SD of micronuclei of lymphocyte blood samples exposed to 2 Gy of 6 Mv-radiation before (0 hour) and after (1, 2, 3 hours) administration of radioprotectors, melatonin and vitamin C, seperately and combined. MN; Micronucli.

The results of this study indicated that the protection efficacy of melatonin is better than vitamin C for irradiated human lymphocytes *in vitro*. However, the best radiation protection efficacy was observed for the simultaneous ingestion of both radioprotective agents. In irradiated samples, a statistically significant difference in the total number of micronuclei was observed between samples treated by melatonin or vitamin C and those treated by the combination of melatonin and vitamin C (P<0.05, Fig.2). 

**Table 1:** The percentage of micronuclei induced by exposure to 2 Gy of 6 MV-radiation in blood lymphocytes at 10 minutes before, and at 1, 2 and 3 hours after administration of melatonin (group A) (P<0.001)


Time	At 0 hour		At 1 hour		At 2 hour		At 3 hour	
Volunteer no.	Control	Irradiated	Control	Irradiated	Control	Irradiated	Control	Irradiated

1	0.6	10.3	0.7	5.3	0.8	8.2	0.6	9.7
2	0.6	11.1	0.8	6.1	0.7	9.4	0.5	11.9
3	0.5	12.2	0.6	7.3	0.4	10.3	0.7	11.5
4	0.7	9.2	0.6	5.7	0.8	7.32	0.7	8.7
5	0.4	10.7	0.6	4.8	0.2	8.1	0.5	9.6


**Table 2:** The percentage of micronuclei induced by exposure to 2 Gy of 6 MV-radiation in blood lymphocytes at 10 minutes before and at 1, 2 and 3 hours after administration of vitamin C (group B) (P<0.001)


Volunteer no.	Control	Irradiated	Control	Irradiated	Control	Irradiated	Control	Irradiated

1	0.5	10.4	0.6	6.6	0.4	9.6	0.7	10.1
2	0.8	13.1	0.7	9.17	0.6	12	0.7	12.4
3	0.6	11.2	0.5	6.9	0.7	9.5	0.8	10.9
4	0.4	10.8	0.5	7.2	0.7	9.8	0.6	11.8
5	0.7	9.7	0.9	5.8	0.8	8.4	1.1	8.8


**Table 3:** The percentage of micronuclei induced by exposure to 2 Gy of 6 MV-radiation in blood lymphocytes at 10 minutes before and at 1, 2 and 3 hours after administration of melatonin and vitamin C (group C) (P<0.005)


Volunteer no.	Control	Irradiated	Control	Irradiated	Control	Irradiated	Control	Irradiated

1	0.5	9.5	0.7	4.1	0.8	6.6	0.9	7.6
2	0.8	11.2	0.7	4.7	0.7	7.9	0.6	9.1
3	0.9	11.8	0.7	5.1	0.8	8.7	0.4	9.7
4	0.8	12.2	0.6	5.2	0.8	8.8	0.7	10.2
5	0.7	10.5	1.1	4.2	0.8	7.5	0.9	8.9


## Discussion

A radioprotector with high efficacy can be used to protect people from unwanted exposure in occupational settings, nuclear accidents, radiotherapyand potentially space travel (17,18). 

Two-thirds of chromosomal damages induced by ionizing radiation are caused by generated free radicals. Antioxidants, due to their ability to scavenge the free radicals, can reduce the unfavorable effects of ionizing radiation on living systems such as chromosomal damages. The ability to scavenge free radicals have been demonstrated in many studies for melatonin and vitamin C (5,19,24). 

The radiation protection efficacy of vitamin C and melatonin was investigated and confirmed, both of which are natural compounds in the human body (24,26). But few human studies have been carried out on these agents simultaneously. In this study, the ability of x-ray radiation to increase the incidence of micronuclei in human lymphocytes was confirmed as previously reported (6,8). Follow-up of all volunteers for two months after participating in this study did not show any side effects for melatonin and vitamin C ingestionin all three groups. In addition, chromosomal abnormalities were reduced significantly after the administration of melatonin, vitamin C and their combination. Moreover, our results show that combined use of melatonin and vitamin C can have a synergistic effect of these compounds. In human studies (*in vitro*), significant decrease in chromosomal damages caused by gamma radiation in the presence of these compounds was shown similarly (4,5,17,18). 

We used 200 cGy of 6 MV irradiation since it is a standard fraction that is administered to patients daily in a fractionation regimen and the highest sensitivity for human DNA is obtained for this dose of radiation (17). The results of this study showed that the maximum radioprotective effect on blood lymphocytes (in all three groups) was obtained 1 hour after melatonin, vitamin C and their simultaneous ingestion and this effect was reduced over time. 

## Conclusion

We conclude that melatonin and vitamin C can reduce DNA damage caused by x-ray photons in human peripheral blood lymphocytes, if given before irradiation. Melatonin and vitamin C are natural compounds in the human body and therefore do not have considerable side effects with usual administration doses. This study contributes to the growing body of evidence on radiation protection efficacy of melatonin and vitamin C and reports that their combination shows a synergistic effect. 

Due to significant differences between *in vitro* and *in vivo* conditions for radiation exposure, administering melatonin and vitamin C as radioprotectors for clinical applications requires further experimental studies by using different cytogenetic assays. Moreover, conjugation of these compounds with a monoclonal antibody could increase the efficacy of these radioprotective compounds for cancer treatment. 

## References

[1] Iyer R, Lehnert BE (2000). Effects of ionizing radiation in targeted and nontargeted cells. Arch Biochem Biophys.

[2] Steenken S (1989). Purine bases, nucleosides, and nucleotides: aqueous solution redox chemistry and transformation reactions of their radical cations and eand OH adducts. Chem Rev.

[3] Hosseinimehr SJ (2007). Trends in the development of radioprotective agents. Drug Discov Today.

[4] Karbownik M, Reiter RJ (2000). Antioxidative effects of melatonin in protection against cellular damage caused by ionizing radiation. Proc Soc Exp Biol Med.

[5] Vijayalaxmi A, Reiter RJ, Tan DX, Herman TS, Thomas CR Jr (2004). Melatonin as a radioprotective agent: a review. Int J Radiat Oncol Biol Phys.

[6] Hardeland R, Pandi-Perumal SR (2005). Melatonin, a potent agent in antioxidative defense: actions as a natural food constituent, gastrointestinal factor, drug and prodrug. Nutr Metab (Lond).

[7] Sharma S, Haldar C, Chaube SK (2008). Effect of exogenous melatonin on X-ray induced cellular toxicity in lymphatic tissue of Indian tropical male squirrel, funambulus pennanti. Int J Radiat Biol.

[8] Shirazi A, Ghobadi G, Ghazi-Khansari M (2007). A radiobiological review on melatonin: a novel radioprotector. J Radiat Res.

[9] Reiter RJ (2000). Melatonin: lowering the high price of free radicals. News Physiol Sci.

[10] Rodriguez C, Mayo JC, Sainz RM, Antolin I, Herrera F, Martin V (2004). Regulation of antioxidant enzymes: a significant role for melatonin. J Pineal Res.

[11] Shirazi A, Mihandoost E, Mohseni M, Ghazi-Khansari M, RabieMahdavi S (2013). Radio-protective effects of melatonin against irradiation-induced oxidative damage in ratperipheral blood. Phys Med.

[12] Karbownik M, Reiter R (2000). Antioxidative effects of melatonin in protection against cellular damage caused by ionizing radiation. Proc Soc Exp Biol Med.

[13] Acuña Castroviejo D, López LC, Escames G, López A, García JA, Reiter RJ (2011). Melatonin-mitochondrial interplay in health and disease. Curr Top Med Chem.

[14] Konopacka M, Rzeszowska-Wolny J (2001). Antioxidant Vitamins C, E and beta-carotene reduce DNA damage before as well as after gamma-ray irradiation of human lymphocytes in vitro. Mutat Res.

[15] Fenech M, Morley AA (1985). Measurement of micronuclei in lymphocytes. Mutat Res.

[16] Brengues M, Liu D, Korn R, Zenhausern F (2014). Method for validating radiobiological samples using a linear accelerator. EPJ Tech Instrum.

[17] Palyvoda O, Polańska J, Wygoda A, Rzeszowska-Wolny J (2003). DNA damage and repair in lymphocytes of normal individuals and cancer patients: studies by the comet assay and micronucleus tests. Acta Biochim Pol.

[18] Menon A, Nair CKK (2011). POLY MVAa dietary supplement containing α-lipoic acid palladium complex, enhances cellular DNA repair. Int J Low Radiat.

[19] Mukherjee D, Roy SG, Bandyopadhyay A, Chattopadhyay A, Basu A, Mitra E (2010). Melatonin protects against isoproterenol-induced myocardial injury in the rat: antioxidative mechanisms. J Pineal Res.

[20] Reiter RJ, Manchester LC, Tan DX (2010). Neurotoxins: free radical mechanisms and melatonin protection. Curr Neuropharmacol.

[21] Vera-Ramirez L, Sanchez-Rovira P, Ramirez-Tortosa MC, Ramirez-Tortosa CL, Granados-Principal S, Lorente JA (2011). Free radicals in breast carcinogenesis, breast cancer progression and cancer stem cells.Biological basis to develop oxidative-based therapies. Crit Rev Oncol Hematol.

[22] Galano A, Tan DX, Reiter RJ (2011). Melatonin as a natural ally against oxidative stress: a physiochemical examination. J Pineal Res.

[23] Yilmaz S, Yilmaz E (2006). Effects of melatonin and vitamin E on oxidative-antioxidative status in rats exposed to irradiation. Toxicology.

[24] Reiter RJ, Tan DX, Manchester LC, Qi W (2001). Biochemical reactivity of melatonin with reactive oxygen and nitrogen species: a review of the evidence. Cell Biochem Biophys.

[25] Mathew D, Nair CK, Jacob JA, Biswas N, Mukherjee T, Kapoor S (2007). Ascorbic acid monoglucoside as antioxidant and radioprotector. J Radiat Res.

[26] Das B, Bennett PV, Cutter NC, Sutherland JC, Sutherland BM (2011). Melatonin protects human cells from clustered DNA damages, killing and acquisition of soft agar growth induced by X-rays or 970 MeV/n Fe ions. Int J Radiat Biol.

